# An in vitro analysis of medial structures and a medial soft tissue reconstruction in a constrained condylar total knee arthroplasty

**DOI:** 10.1007/s00167-016-4087-0

**Published:** 2016-03-29

**Authors:** Kiron K. Athwal, Hadi El Daou, Eivind Inderhaug, William Manning, Andrew J. Davies, David J. Deehan, Andrew A. Amis

**Affiliations:** 1 Department of Mechanical Engineering, Imperial College London, Exhibition Road, London, SW7 2AZ UK; 2 Guy’s Hospital, Great Maze Pond, London, SE1 9RT UK; 3 Department of Orthopaedic Surgery, Newcastle Freeman University Hospital, Newcastle upon Tyne, UK; 4 Musculoskeletal Surgery Group, Department of Surgery and Cancer, Charing Cross Hospital, Imperial College London School of Medicine, London, W6 8RF UK

**Keywords:** Knee replacement, Constrained implant, Reconstruction, Total knee arthroplasty, Medial collateral ligament, Soft tissue deficiency, Stability, Laxity

## Abstract

**Purpose:**

The aim of this study was to quantify the medial soft tissue contributions to stability following constrained condylar (CC) total knee arthroplasty (TKA) and determine whether a medial reconstruction could restore stability to a soft tissue-deficient, CC-TKA knee.

**Methods:**

Eight cadaveric knees were mounted in a robotic system and tested at 0°, 30°, 60°, and 90° of flexion with ±50 N anterior–posterior force, ±8 Nm varus–valgus, and ±5 Nm internal–external torque. The deep and superficial medial collateral ligaments (dMCL, sMCL) and posteromedial capsule (PMC) were transected and their relative contributions to stabilising the applied loads were quantified. After complete medial soft tissue transection, a reconstruction using a semitendinosus tendon graft was performed, and the effect on kinematic behaviour under equivocal conditions was measured.

**Results:**

In the CC-TKA knee, the sMCL was the major medial restraint in anterior drawer, internal–external, and valgus rotation. No significant differences were found between the rotational laxities of the reconstructed knee to the pre-deficient state for the arc of motion examined. The relative contribution of the reconstruction was higher in valgus rotation at 60° than the sMCL; otherwise, the contribution of the reconstruction was similar to that of the sMCL.

**Conclusion:**

There is contention whether a CC-TKA can function with medial deficiency or more constraint is required. This work has shown that a CC-TKA may not provide enough stability with an absent sMCL. However, in such cases, combining the CC-TKA with a medial soft tissue reconstruction may be considered as an alternative to a hinged implant.

## Introduction

Constrained condylar total knee arthroplasty (CC-TKA) was introduced as a form of knee replacement that offers more stability than conventional posterior-stabilised (PS) TKA [36]. The larger post-cam mechanism is intended to provide more support in varus–valgus and minimise risk of posterior dislocation of the post. Therefore, it has been utilised when a primary TKA fails and requires revision [6], or as a more constrained primary choice if the surgeon is unable to balance the knee in both flexion and extension [18].

Further restraint can be found in a rotating hinge (RH) design with linked tibial and femoral components. However, as the design becomes more constrained and massive, greater loads are transmitted to the implant–bone interface (with less soft tissue support), and thus loosening becomes a greater risk [3], as well as requiring more bone resection [23]. According to the manufacturer’s indications for the implant used in this study (P.F.C. Sigma TC3; DePuy Synthes Joint Reconstruction, Leeds, UK) [7], an RH implant should be used instead of a CC implant when the medial collateral ligament (MCL) is absent. Many papers support the use of CC over PS-TKA when the MCL is present and lax, and the use of a RH over CC-TKA with absence of the MCL [10, 11, 23, 32]. This is not always followed clinically as surgeons would prefer to minimise size and constraint of the implant and still achieve stability. There is little detailed evidence to show how much CC-TKA relies on the soft tissue to provide constraint, and therefore, it is difficult to inform the surgeon when a CC-TKA would be preferable to a PS-TKA, or when to move from a CC-TKA to a RH design.

MCL injury is common in sports [4]; therefore, there are many suggested medial reconstructions [15, 20, 21, 41]. This introduces the option of a medial reconstruction in conjunction with a CC-TKA in younger patients with medial soft tissue deficiency, instead of revising to a more constrained RH [22, 23, 25, 35]. The advantages would be avoiding higher fixation stresses and larger loss of bone stock inherent with RH-TKA, although Morgan et al. [23] also highlighted problems including increased surgical time, difficult flexion/extension balancing of the knee, and donor site morbidity with autografts. Despite studies reporting mixed results in TKA patients with medial reconstructions, MCL advancement to tighten the ligament, or repair to iatrogenic injury [12, 17, 26, 28, 33], there have been no biomechanical studies assessing the feasibility of using a medial soft tissue reconstruction in a TKA implanted knee to restore kinematics to intact medial soft tissue conditions.

The aim of this study was to measure the relative contributions of medial soft tissue structures in a CC implanted knee and for the first time the biomechanics of a medial reconstruction with implanted knees. This will allow clinicians to better understand the indications for constrained implant use in presence of ligamentous deficiency, as well as determine how well a medial reconstruction functions in conjunction with implants. The null hypotheses were: (1) that there would be no significant difference in the relative contributions of each of the medial soft tissue structures to resisting tibiofemoral laxity and (2) that the surgical reconstruction of the medial soft tissue structures would restore pre-sectioned kinematics.

## Materials and methods

Following ethics approval, eight fresh-frozen human cadaver (six male and two female) knee specimens of median age 68 (range 63–72) were obtained from a tissue bank (five right-sided and three left-sided). The knees had no evidence of fixed flexion, misalignment, or significant arthritic deterioration.

For each specimen a midline incision was performed, followed by a medial parapatellar arthrotomy. A rotating-platform CC-TKA (P.F.C. Sigma TC3; DePuy Synthes Joint Reconstruction, Leeds, UK) was implanted by a consultant orthopaedic surgeon, using a standard combination of measured resection and gap balancing at full extension and 90° flexion. The femur was referenced using an intramedullary guide rod set at 5° of valgus. The distal femoral cutting block was placed in neutral rotation with respect to the epicondylar axis, and a 9-mm distal femur resection measured from the most prominent condyle. Femoral sizing was performed using an anterior down technique. The tibia was referenced using an intramedullary rod with a 3° posterior slope on the tibial block positioned with respect to the tibial anterior prominence, corresponding to the centre of the tibial tuberosity in our knee specimens. From the most superior proximal tibial surface, 10 mm of bone was resected. Gap balancing was confirmed using spacers to achieve a rectangular space both in full extension and flexion after bone resection but before chamfer femoral cuts. Both tibial and femoral components were cemented to the bone using polymethyl methacrylate (PMMA). No soft tissue releases were performed and ‘tenting’ of the collateral ligaments was avoided by removing any osteophytes. A stable knee was taken as that which allowed for unimpeded tracking of the patella and no medial or lateral opening after implant trialling, throughout a passive flexion arc from full extension.

### Robotic biomechanical testing system

The specimens were mounted in a robotic testing system, comprising of a six-degree-of-freedom (DOF) robotic manipulator and controller (TX90 and CS8C; Stäubli Ltd, Zürich, Switzerland), and a six-axis force/torque sensor (Omega 85; ATI Industrial Automation, Apex NC). The manipulator had a maximum load of 200 N and repeatability of 0.03 mm in translation (manufacturer’s specification). The sensor had a force sensing range of 3800 N (resolution ± 0.43 N) for the *Z* axis and 1900 N (resolution ± 0.29 N) for the *X* and *Y* axes, and torque sensing range of 80 Nm for *Z*, *X*, and *Y* axes (*Z* resolution ± 0.009 Nm and *X*–*Y* resolution ± 0.013 Nm), with an accuracy of ±1 N and ±0.1 Nm. The femur was fixed rigidly to the base of the robot, and the tibia was attached to the force sensor on the end-effector of the manipulator.

To prepare the knee for robotic testing, the tibia/fibula and femur were skeletonised 80 and 110 mm from the joint line, respectively. The head of the fibula was fixed to the tibia using a transcortical screw to maintain its anatomical position. The femur and tibia were fixed in 60-mm-diameter cylindrical steel pots using PMMA. The tibia was aligned centrally in the bone pot using a jig with a pointer that located the centre of the tibia through the medial parapatellar arthrotomy as between the tips of the tibial spines [1]. The femur was cemented in the bone pot whilst in full extension, with the posterior condylar axis aligned parallel to the femoral fixture, and the arthrotomy was sutured.

### Testing protocol

The knee was manually flexed 20 times to minimise soft tissue hysteresis, and then in the robot a path of passive flexion from 0° to 90° was performed. The robotic system minimised forces and moments in the other five DOFs acting across the knee and recorded the position of the knee at 0°, 30°, 60°, and 90° of flexion, which were the starting points for the following loads to the tibia: ±50 N anterior–posterior (AP) force, ±8 Nm varus–valgus (VV) torque, and ±5 Nm internal–external (IE) rotational torque. In each test, the robotic system applied the force/torque in the primary DOF, maintained the same flexion/extension DOF, and minimised the loads in the four remaining DOF. These loads were comparable to other studies of intraoperative laxity measurement [27, 29, 31], and each test was repeated three times; the muscles were not tensed.

Whilst the knee remained attached to the robot, the deep MCL (dMCL), the anterior fibres of the superficial MCL (sMCL), the complete sMCL, and the posteromedial capsule (PMC) were sequentially released (Table 1). The dMCL was transected with a scalpel deep to the ligament and just distally to the joint line, whilst also removing any meniscus and connected tissues. The anterior fibres of the sMCL (as defined by Whiteside et al. [38, 39]) were released from their distal tibial attachment subperiosteally using an osteotome, and the remaining fibres were transected at the joint line as the second stage. The PMC fibres attached to the semimembranosus tendon were transected posteromedially from the dMCL. After each structure was transected/released and the arthrotomy was resutured, the robot reproduced the kinematics from the initial stage to calculate the force/moment restraint offered by each structure cut using the principle of superposition [30].

**Table 1:** Outline of the experimental protocol and data obtained

Knee state	Kinematic test	Data obtained
Implanted knee	±50 N AP, ±8 Nm VV, ±5 Nm IE	Kinematics of implant knee (I)
Transect dMCL	Repeat kinematics I	Restraining force/moments from dMCL
Release anterior fibres of sMCL	Repeat kinematics I	Restraining force/moments from anterior fibres of sMCL
Transect entire sMCL	Repeat kinematics I	Restraining force/moments from sMCL
Transect PMC	Repeat kinematics I	Restraining force/moments from PMC
Reconstruction	±50 N AP, ±8 Nm VV, ±5 Nm IE	Kinematics of reconstructed knee (II)
Release reconstruction	Repeat kinematics II	Restraining force/moments from reconstruction

*AP* anterior–posterior force, *VV* varus–valgus torque, *IE* internal–external torque, *dMCL* deep medial collateral ligament, *sMCL* superficial medial ligament, *PMC* posterior medial capsule

### Reconstruction

**Fig. 1:**
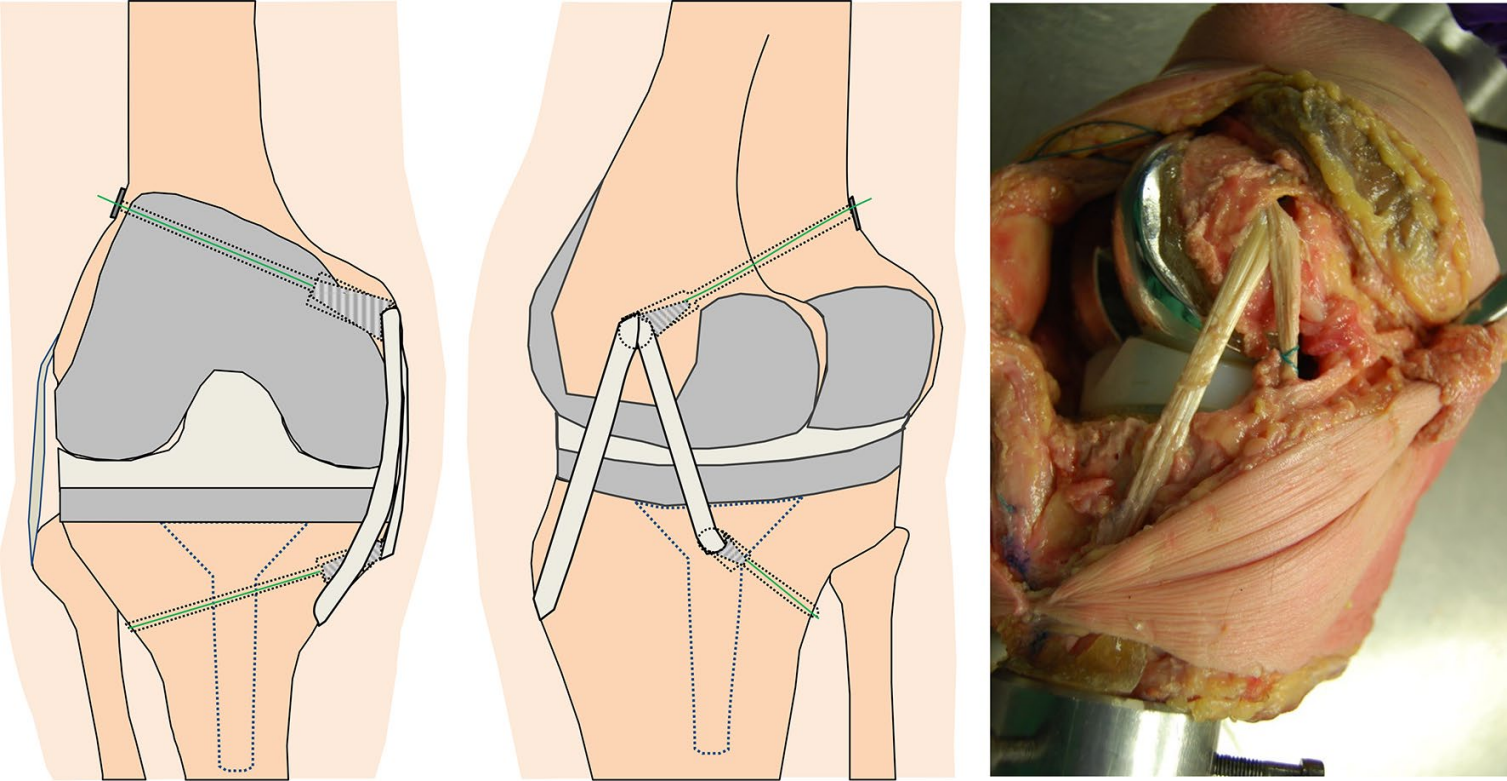
Graphic representation of the medial reconstruction in coronal (*left*) and posteromedial (*central*) views and in a cadaveric specimen (*right*)

After the soft tissues were cut and tested, the knee was removed from the robot, and a medial soft tissue reconstruction, similar to Lind et al. [21], was performed (Fig. 1). The semitendinosus tendon was harvested but kept attached to the pes anserinus insertion. The free end of the tendon was whip-stitched with a size 2 Ethibond Excel suture (Ethicon Inc, Somerville, NJ). An 8-mm tunnel was drilled on the posteromedial aspect of the tibia, and the free end of the graft was passed into the tunnel and secured with a 9-mm metal interference screw. An 8-mm femoral tunnel was placed just anterior to the most prominent point of the medial epicondyle (this point was found to reduce impingement with the remnants of the MCL). The isometry of this point was tested during extension and flexion, and if the anterior arm of the reconstruction was judged to have excessive tension during knee flexion, a more posterior tunnel placement was tested. After graft passage, whilst holding the knee in neutral tibial rotation and varus torque to reduce medial gapping [40] at 45° flexion, the graft was tensioned to 20 N (This tension was found in pilot testing) and fixed to the contralateral cortex with a 20-mm EndoButton (Smith & Nephew Inc, Memphis, TN). At the medial femoral aperture, the graft was secured with an additional 9-mm metal interference screw.

The reconstructed knee was manually flexed three times to minimise hysteresis, and then the femur was clamped in a vice at 0° and 90° flexion. A spring balance applied a lateral force to the tibial shaft to impose 8 Nm valgus torque on the joint. The resulting medial gap opening was converted into a valgus rotation and compared with the laxity of a previous study [2] to ensure that it was within the native range.

The reconstructed knee was then remounted on the robot, and the loading protocol was repeated. Finally, the reconstruction was released from its femoral tunnel, and the loading protocol was repeated again, to calculate the force/moment restraint offered by the reconstruction (Table 1).

Approval for this study (project code R13066) was given by the Imperial College Healthcare Tissue Bank under the Human Tissue Authority licence number 12275.

### Statistical analysis

Mean peak forces/torques, translations/rotations, and soft tissue contributions (the drop in force/torque after the transection/release as a percentage of the original force/torque value) were calculated using a custom MATLAB (MathWorks, Natick, MA) script. Output data were determined to one decimal place because the system repeatability was 0.03 mm; the percentage results are quoted to the nearest whole number. The following statistical analyses were performed in SPSS 22 (IBM SPSS Statistics, version 22, Armonk, NY):

1. Two-way repeated-measures analysis of variance (RM-ANOVA) was performed to compare laxities to the knee state (intact and reconstructed knee) across different flexion angles.
2. One-way RM-ANOVA was performed at each flexion angle to compare the force/torque contribution to the medial structure cut.
3. One-way RM-ANOVA was performed at each flexion angle to compare between the force/torque contribution of the reconstruction and the native sMCL, and between the reconstruction and the total native medial complex (dMCL, sMCL and PMC).

For all analyses, post hoc *t* tests with Bonferroni correction were performed when differences were found, and the significance level was set at *p* < 0.05. A power calculation, based on a mean change in translation of 3.5 ± 3 mm and rotation of 3.7 ± 3.2 in a prior study [2], determined a sample size of eight would be needed to detect a significant laxity change and soft tissue contributions of 9 % with 80 % power and 95 % confidence. Therefore, a significant restraint for a given flexion angle was defined as having a statistically significant mean resisting contribution greater than a threshold value of 10 %.

## Results

### Valgus rotation

**Fig. 2:**
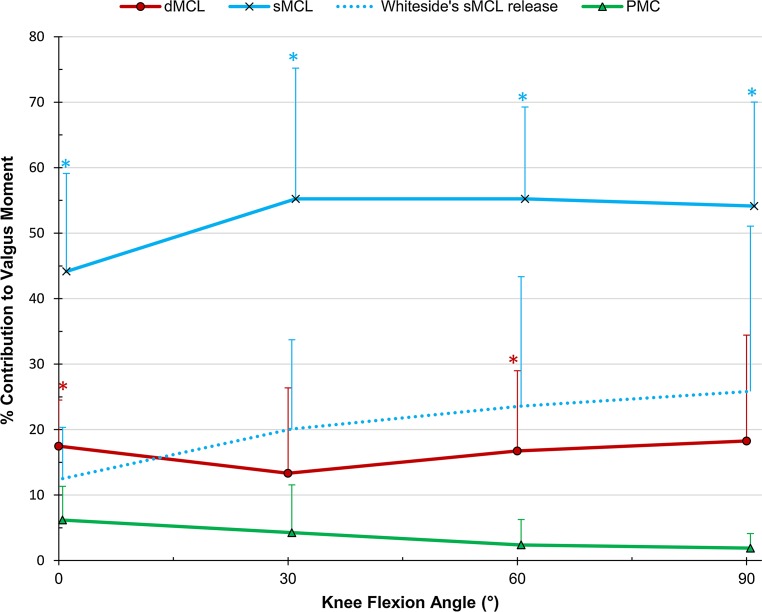
Percentage contributions of the deep and superficial medial collateral ligaments (dMCL and sMCL) and posteromedial capsule (PMC) in resisting 8 Nm valgus moment in implanted knees, Mean ± SD. *Asterisk* indicates a statistically significant contribution greater than 10 % at the specified flexion angle (*p* < 0.05)

**Fig. 3:**
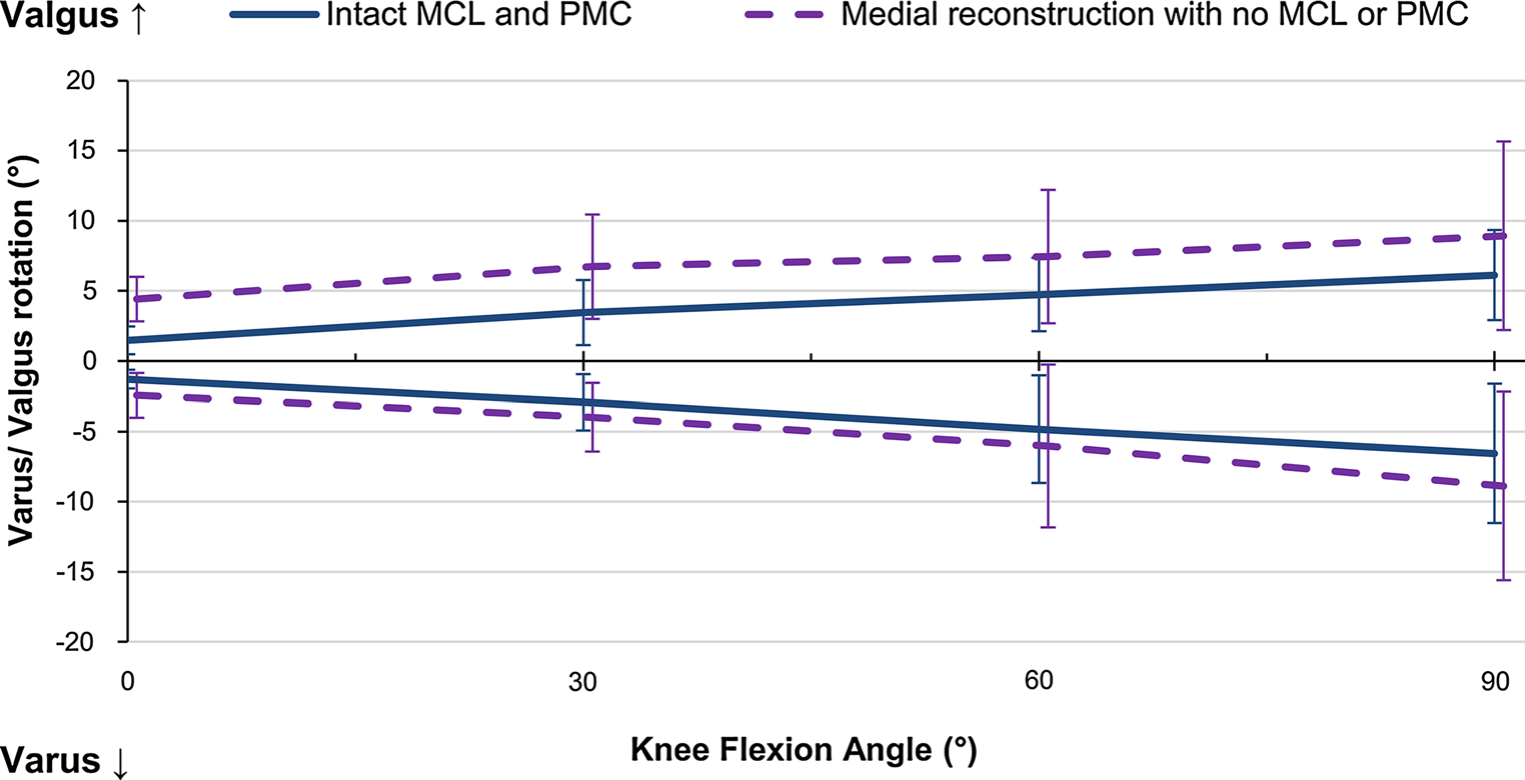
Varus–valgus rotation of implanted knees with intact medial complex, and the same knees with the medial complex transected and a medial reconstruction, in response to a ± 8 Nm varus–valgus moment. Mean ± SD at each flexion angle. *MCL* medial collateral ligament (both superficial and deep), *PMC* posteromedial capsule

The sMCL was the primary restraint, resisting 52 % on average across flexion angles (Fig. 2). The dMCL was a significant restraint at 0° and 60° (17 ± 7 and 17 ± 12 %, *p* = 0.002 and 0.033, respectively). The reconstruction restraint was significantly lower than the combined medial contributions at 0° flexion; however, at 60° the reconstruction was significantly higher than the individual sMCL contribution (Table 2). There was no significant interaction between flexion angle and intact/reconstructed state (Fig. 3). Release of the anterior sMCL fibres reduced the restraint by 13 ± 8 % (n.s.) at 0° to 26 ± 25 % (n.s.) at 90° flexion; however, this was not significant. The released fibres were also not a significant restraint under AP or IE loads.

**Table 2:** Relative contributions of the reconstruction, superficial medial collateral ligament and combined medial structures to the applied loads

Flexion angle	Contribution to varus (%)			Contribution to valgus (%)		
	Reconstruction	sMCL	sMCL, dMCL and PMC	Reconstruction	sMCL	sMCL, dMCL and PMC
Varus/valgus tibial torque ± 8 Nm						
0°	7 ± 7	11 ± 12	20 ± 15^R^	40 ± 20	44 ± 15	66 ± 16^R^
30°	5 ± 5	7 ± 5	15 ± 8^R^	70 ± 6	55 ± 20	71 ± 16
60°	6 ± 5	8 ± 4	15 ± 7^R^	76 ± 9^S^	55 ± 14	72 ± 10
90°	4 ± 4	7 ± 5	10 ± 6^R^	71 ± 16	54 ± 16	72 ± 14
Anterior/posterior tibial force ± 50 N						
0°	17 ± 19	29 ± 7	47 ± 19^R^	11 ± 13	17 ± 9	22 ± 9
30°	34 ± 22	35 ± 19	52 ± 18^R^	22 ± 21	17 ± 14	24 ± 18
60°	41 ± 24	33 ± 20	50 ± 27^R^	22 ± 21	13 ± 15	28 ± 13
90°	41 ± 29	34 ± 22	50 ± 30	15 ± 9	10 ± 8	18 ± 7
Internal/external tibial torque ± 5 Nm						
0°	26 ± 17	22 ± 15	42 ± 19^R^	13 ± 11	30 ± 12	47 ± 13^R^
30°	40 ± 15	29 ± 17	50 ± 18	28 ± 11	27 ± 14	45 ± 13^R^
60°	45 ± 10	30 ± 16	48 ± 18	37 ± 13	28 ± 14	46 ± 14
90°	38 ± 19	31 ± 16	46 ± 16	41 ± 17	30 ± 15	46 ± 13

*dMCL* deep medial collateral ligament, *sMCL* superficial medial collateral ligament, *PMC* posterior medial capsule

^R^ Significant difference between reconstruction and combined sMCL, dMCL and PMC contributions (*p* < 0.05)

^S^ Significant difference between reconstruction and sMCL contributions (*p* < 0.05)

### Varus rotation

None of the medial structures sectioned had a significant contribution above 10 %. There was also no significant two-way interaction between flexion angle and intact/reconstructed state.

### Anterior translation

**Fig. 4:**
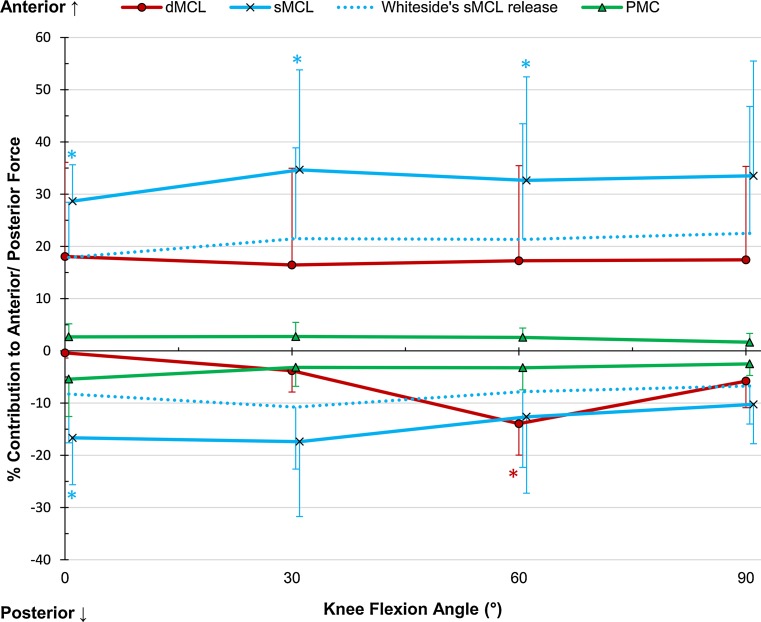
Percentage contributions of the deep and superficial medial collateral ligaments (dMCL and sMCL) and posteromedial capsule (PMC) in resisting ± 50 N anterior–posterior force in implanted knees, Mean ± SD. *Asterisk* indicates a statistically significant contribution greater than 10 % at the specified flexion angle (*p* < 0.05)

**Fig. 5:**
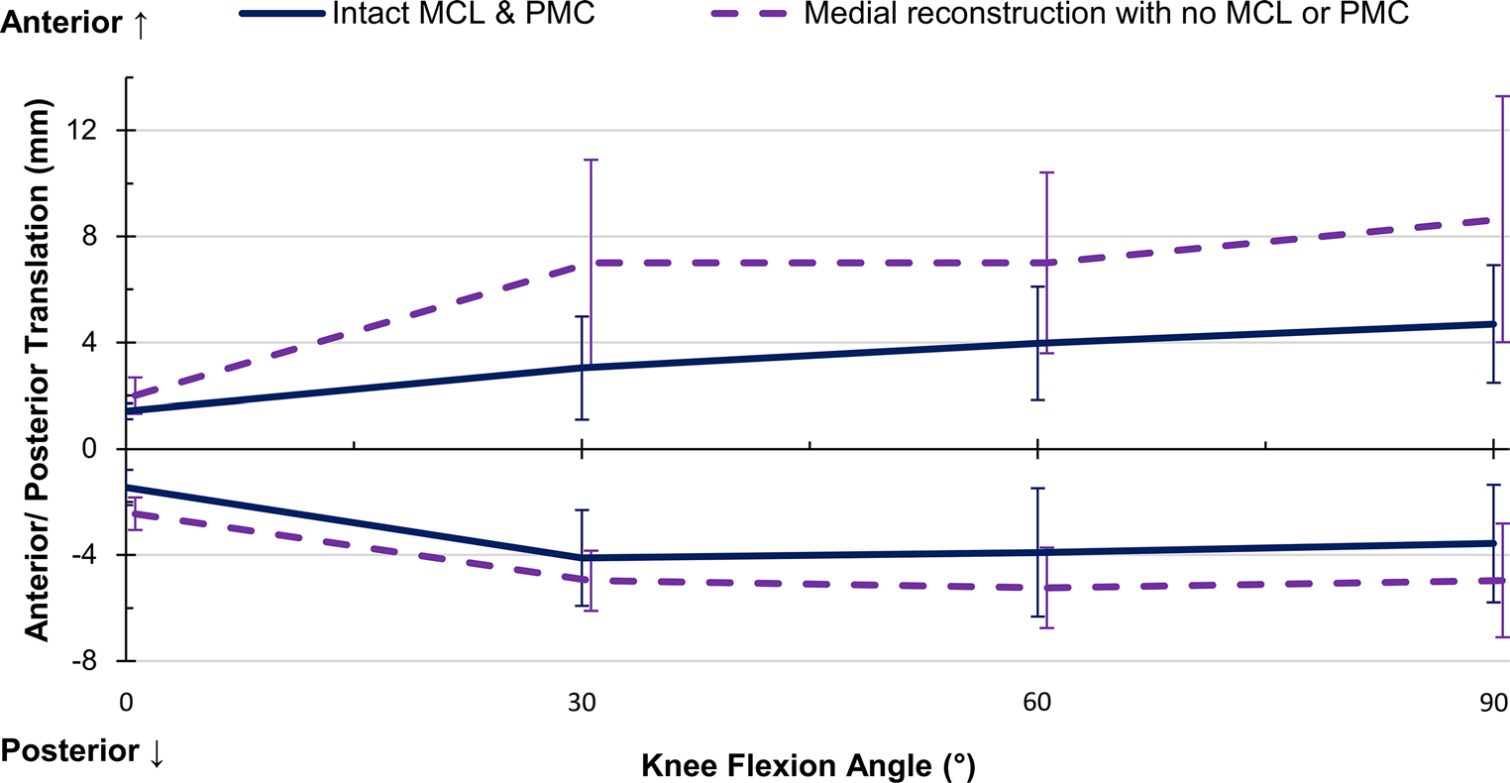
Comparison of anterior–posterior translation of **a** implanted knees with intact medial complex, and **b** the same knees with medial complex transected and a medial reconstruction, in response to a ± 50 N anterior–posterior force. *Error bars* denote the standard deviation at each flexion angle. *MCL* medial collateral ligament (both superficial and deep), *PMC* posteromedial capsule

The only significant restraint was the sMCL at 0°, 30°, and 60° (*p* = 0.001, 0.014, and 0.022, respectively), which contributed on average 32 % across flexion angles (Fig. 4). The reconstruction restraint was significantly smaller than the combined medial contributions at 0°, 30°, and 60°, but no significant difference was found between the reconstruction and the sMCL restraint alone (Table 2). No significant difference was found in the two-way interaction between flexion angle and intact/reconstructed state (Fig. 5); however, paired *t* tests at 90° found the reconstructed translation to be larger than the native state (*p* = 0.037).

### Posterior translation

The sMCL restrained 17 ± 9 % at 0° (*p* = 0.020) and the dMCL 14 ± 6 % at 60° (*p* = 0.013, Fig. 4). The reconstruction was not significantly different from either the sMCL or combined medial contribution (Table 2), and there was no significant two-way interaction between flexion angle and intact/reconstructed state (Fig. 5),

### Internal rotation

**Fig. 6:**
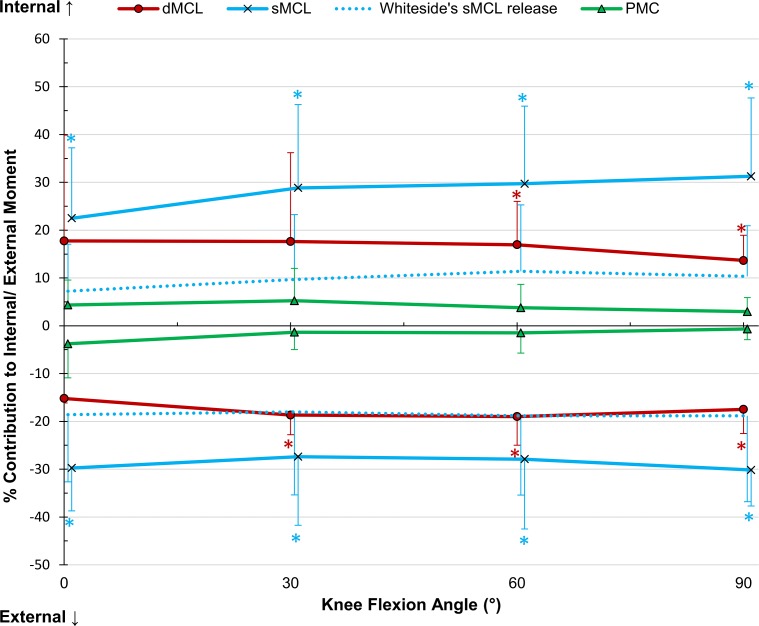
Percentage contributions of the deep and superficial medial collateral ligaments (dMCL and sMCL) and posteromedial capsule (PMC) in resisting ± 5 Nm internal–external moment in implanted knees, Mean ± SD. *Asterisk* indicates a statistically significant contribution greater than 10 % at the specified flexion angle (*p* < 0.05)

**Fig. 7:**
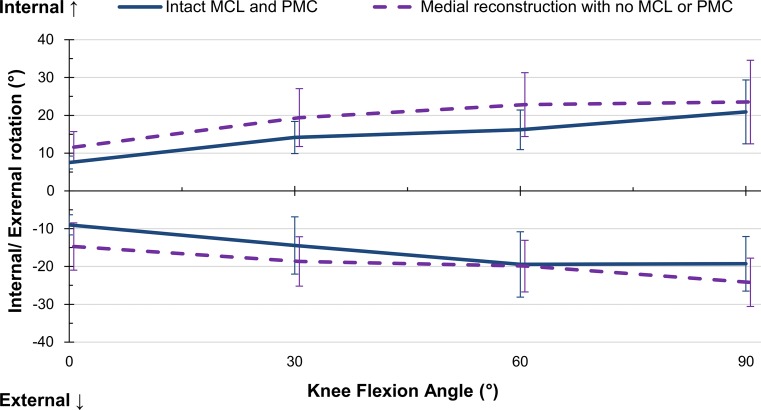
Comparison of internal–external rotation of **a** implanted knees with intact medial complex, and **b** the same knees with medial complex transected and a medial reconstruction, in response to a ± 5 Nm internal–external moment. Mean ± SD. *MCL* medial collateral ligament (both superficial and deep), *PMC* posteromedial capsule

The sMCL was significant at all flexion angles, with an average restraint of 28 % (Fig. 6). The dMCL resisted 17 ± 9 % at 60° (*p* = 0.008) and 14 ± 5 % at 90° (*p* = 0.004). The reconstruction restraint was lower than the combined medial contribution at 0°, however at all other angles no significant difference was found between the reconstruction and the sMCL or combined restraints (Table 2). There was no significant two-way interaction between flexion angle and intact/reconstructed state (Fig. 7).

### External rotation

The sMCL significantly restrained an average of 29 % at all flexion angles (Fig. 6). The dMCL restrained 19 ± 13, 19 ± 11, and 17 ± 8 % at 30°, 60°, and 90°, respectively (*p* = 0.026, 0.011, 0.011). The reconstruction restraint was significantly lower than the combined medial contribution at 30°, but not of the sMCL alone (Table 2). No significant two-way interaction between flexion angle and intact/reconstructed state was found (Fig. 7).

## Discussion

The most important finding of the study was that the sMCL was the primary medial ligamentous restraint to valgus, internal–external rotations, and anterior translation in the CC-TKA knee. The reliance of the CC-TKA on the sMCL indicates that a knee with gross sMCL deficiency would be better served with a hinged implant. Another important finding was that a medial reconstruction used simultaneously with a CC-TKA restored laxity to pre-sectioning values. The relative contribution of the reconstruction was not found to be significantly larger than the sMCL in most cases, and thus it would not be overloaded even with extensive medial deficiency and may be considered as an alternative to a hinged implant.

This study found that an average of 52 % of restraint to an 8 Nm valgus moment was provided by the sMCL. A previous study with PS-TKA found the sMCL to resist an average of 62 % of a valgus moment [2], thus implying the CC-TKA would not compensate for a complete loss of the sMCL. The sMCL also restrained anterior drawer and internal–external rotation, as in other studies of intact and ACL-deficient cadaveric, non-implanted knees [14, 29, 37]. This suggests that, while the CC-TKA can work with a lax sMCL, it would require a RH-TKA when the MCL is completely deficient. Many prospective studies have satisfactory outcomes with MCL incompetency as an indication for CC-TKA as a primary or revision surgery [16, 18, 19]. Vasso et al. [34] did a prospective study to form a selection algorithm for knee revision, and concluded that CC-TKA was suitable for collateral ligament insufficiency but not absence, and hinged implants for complete ligament absence, which agrees with our findings. This is further supported in other reviews [11, 22, 32].

Although the entire sMCL was the primary restraint to valgus rotation at all flexion angles, release of the anterior fibres alone did not significantly reduce restraint of valgus rotation. The large variability between the specimens (Fig. 2) may be explained by the use of the same 9-mm osteotome, which would have elevated different proportions of the total sMCL attachment depending upon the sizes of the knees.

The loads applied in this study were similar to other in vitro TKA studies, yielding similar laxities [2, 24]. Saeki et al. [31] found that under 35 N AP force, 10 Nm VV, and 1.5 Nm IE torques, MCL release increased laxity in cruciate-retaining TKA knees throughout flexion. In studies involving cadaveric knees stressed to a subjective ‘maximum’ displacement/rotation [8, 13], the laxities were comparable to those in this study. Therefore, the loads applied with the robotic system represent a clinical intraoperative assessment of ligament balancing.

Medial soft tissue reconstructions in the native knee have been described. LaPrade et al. used separate grafts for the sMCL and the posterior oblique ligament (POL), with the sMCL graft sutured to the anterior arm of the semimembranosus to recreate a proximal tibial attachment [20]. In a cadaveric study, the reconstructed knee was not significantly different to the intact knee in external rotation and posterior drawer, although there was a small increase in anterior drawer in flexion and in valgus rotation at 0° [5]. Lind et al. described a technique similar to this study with encouraging 24 months of patient follow-up results [21]. A cadaveric robotic study of isolated sMCL repair/reconstruction found reduced laxity compared to the sMCL-sectioned state and restored intact valgus and internal rotations at 60–90° flexion [40].

In this study the reconstruction was tightened at mid-flexion, but it was difficult to get the right balance in flexion and extension. Additionally, the anterior laxity of the reconstructed knee was larger than the intact state at 90° flexion. This suggests that in implanted knees the anterior arm of the reconstruction was not well aligned to provide anterior restraint, despite providing adequate rotational restraint. There is a current trend towards ‘anatomic reconstructions’ to restore normal knee biomechanics, which may need to be reconsidered in TKA patients with different implant shapes and constraints which may alter kinematics. For example, Ghosh et al. [9] found that length changes of the collateral ligaments varied between native and post-TKA states, so reconstructions that have been optimised in tension for native knees may perform differently in TKA knees.

There are limitations with in vitro studies, which include testing at time zero. This cannot take into account biological repair to aid the reconstruction fixation, or how repeated loading may cause graft relaxation and loosening. The effect of healing of released soft tissues back to their attachment to the bone also could not be replicated; however, the study provided an accurate representation of preoperative ligament balancing. The applied forces/torques simulated clinical joint laxity evaluation when the patient lies supine with relaxed muscles, thus investigating the passive contributions of the medial structures. Whereas joint evaluation is performed as a subjective measure by the clinician, this in vitro study provides a repeatable controlled study to investigate soft tissue contributions; however, caution must be applied when extrapolating the results to smaller or higher loads.

This study has for the first time fully delineated the function of the medial soft tissues in the constrained TKA under simulated clinical joint evaluation, as well as the first biomechanical study into soft tissue reconstructions with implants. This work argues that in clinical practice the CC-TKA does not provide enough stability with an absent sMCL; however, a surgeon may consider a soft tissue reconstruction as a viable alternative to a hinged implant with more associated bone resection and higher bone-implant interface stresses [ 22 ]. Future research to optimise tunnel placement and surgical technique will allow the reconstruction to provide the best stability throughout flexion. Prospective clinical studies should be devised to evaluate the approach, which may allow more patients to avoid the bone resection required for a hinged prosthesis.

## Conclusion

This work has determined the relative contributions of medial structures to stability of CC implanted knees, and supports the argument for preservation of the sMCL even in the semi-constrained TKA. If the MCL is deficient, then either a RH-TKA may be appropriate or medial soft tissue repair with a reconstruction may be alternatively considered.
